# Exploring phage–host interactions in *Burkholderia cepacia* complex bacterium to reveal host factors and phage resistance genes using CRISPRi functional genomics and transcriptomics

**DOI:** 10.1128/spectrum.01936-25

**Published:** 2025-10-02

**Authors:** Ben Diaz, Rohan Krishna, Joseph S. Schoeniger, Catherine M. Mageeney

**Affiliations:** 1 Biotechnology & Bioengineering, Sandia National Laboratories 111651, Livermore, California, USA; 2 Biological, Radiation, and Signature Science, Technology, & Engineering Center, Sandia National Laboratories, Livermore, California, USA; Barnard College, Columbia University, New York, New York, USA

**Keywords:** *Burkholderia*, phage–host interaction, bacteriophage

## Abstract

Complex interactions of bacteriophages with their bacterial hosts determine phage host range and infectivity. While phage defense systems and host factors have been identified in model bacteria, they remain challenging to predict in non-model bacteria. In this paper, we integrate functional genomics and transcriptomics to investigate phage–host interactions, revealing active phage resistance and host factor genes in *Burkholderia cenocepacia* K56-2. *Burkholderia cepacia* complex species are commonly found in soil and are opportunistic pathogens in immunocompromised patients. We studied infection of *B. cenocepacia* K56-2 with Bcep176, a temperate phage isolated from *Burkholderia multivorans*. A genome-wide dCas9 knockdown library targeting *B. cenocepacia* K56-2 was constructed, and a pooled infection experiment identified 63 novel genes or operons coding for candidate host factors or phage resistance genes. The activities of a subset of candidate host factor and resistance genes were validated via single-gene knockdowns. Transcriptomics of *B. cenocepacia K56-2* during Bcep176 infection revealed that expression of genes coding for host factor and resistance candidates identified in this screen was significantly altered during infection by 4 h post-infection. Identifying which bacterial genes are involved in phage infection is important to understand the ecological niches of *B. cenocepacia* and its phages*,* and for designing phage therapies.

**IMPORTANCE:**

*Burkholderia cepacia* complex bacteria are opportunistic pathogens inherently resistant to antibiotics, and phage therapy is a promising alternative treatment for chronically infected patients. *Burkholderia* bacteria are also ubiquitous in soil microbiomes. To develop improved phage therapies for pathogenic *Burkholderia* bacteria, or engineer phages for applications, such as microbiome editing, it’s essential to know the bacterial host factors required by the phage to kill bacteria, as well as how the bacteria prevent phage infection. This work identified 65 genes involved in phage–host interactions in *Burkholderia cenocepacia* K56-2 and tracked their expression during infection. These findings establish a knowledge base to select and engineer phages infecting or transducing *Burkholderia* bacteria.

## INTRODUCTION

Viruses that infect bacteria (phages) are the most abundant entities on the planet (1, 2), found everywhere, including soil and marine ecosystems (3), ancient sediment (4), wastewater (5), and bioprocessing facilities (6). While their large role in the ecology of microbial communities has started to be appreciated only recently (3, 7, 8), their intrinsic capacity to infect and kill bacterial cells has been studied by biologists for over 100 years (9). Phages are major agents of horizontal gene transfer via transduction (10, 11), contributing to the host pan-genome diversity (12). Their use in treating bacterial infections in economically important plants (13) and in humans (phage therapy [14]) is increasingly viable, especially as antibiotic resistance levels rise globally (15).

Typical strategies bacteria employ to prevent productive phage infection include bacterial encoded defense systems (e.g., restriction endonucleases or CRISPR) that attack the phage’s genome (16). Many bacterial defense mechanisms have been discovered and described in model bacteria/phage combinations, and knowledge is growing rapidly with modern high-throughput methods (16). Bacteria can also evolve under viral selection pressure to deplete or mutate host factors, the bacterially-encoded genes essential for phage infection. Bacterial host factors for phage infection have mainly been described in model bacteria/phage combinations (e.g., *Escherichia coli, Staphylococcus aureus, Salmonella enterica, Pseudomonas aeruginosa,* and *Bacillus subtilis*) and include genes involved in nucleotide biosynthesis/modification, DNA and RNA polymerases, and ribosomes (17–21). Extracellular bacterial cofactors or environmental factors, such as divalent cations, are also known to affect phage infectivity (22, 23). To further understanding of phage interactions in natural environments and bring phage biotechnology applications to maturity, it’s necessary to expand knowledge of phage-host interactions to uncover novel defense systems and host co-factors in non-model bacterial hosts.

*Burkholderia* is a diverse genus whose hallmark is genomic plasticity and rapid acquisition and maintenance of resistance to antibiotics (24). *Burkholderia* phage–host interaction studies may improve our knowledge of mechanisms for rapid acquisition of phage resistance traits, improve host annotation, and discover new tools for synthetic biology. Most work to understand *Burkholderia* physiology and develop genetic tools has been done in opportunistic pathogen strains within the *Burkholderia cepacia* complex (BCC) strains (25, 26). Many *Burkholderia*, including some BCC strains (*B. vietnamensis*) and other highly pathogenic *Burkholderia* species (*B. pseudomallei* and *B. mallei*), are common soil microbes globally (27–29). Additionally, some of these soil-dwelling strains promote plant growth and bioremediation (30–32). Thus, improved knowledge of phage–host interactions may enable phage applications in human therapeutics, soil and rhizosphere health, and bioremediation (26). However, only a few phages have been described that infect *Burkholderia* species (26) presenting an opportunity to characterize phage–host interactions in *Burkholderia* as more phages are discovered. This present study seeks to use a multi-omics approach to deepen our understanding of the host genes involved during phage infection in the BCC.

Here, we focus on phage–host interactions between *Burkholderia cenocepacia* K56-2, isolated originally from a chronically infected cystic fibrosis patient’s sputum (33), and Bcep176, a temperate phage induced from *Burkholderia multivorans*. We utilized two approaches to understand the host’s (K56-2) response to Bcep176 phage infection: (i) transcriptomes of Bcep176-infected *B. cenocepacia* K56-2 to establish the host’s transcriptional response to phage infection, and (ii) a CRISPRi knockdown library screen to identify novel host factors and phage resistance gene candidates. Combining the two approaches allowed us a unique time-resolved view of bacterial response to phage infection.

## RESULTS

### Bcep176 infection kinetics

**Fig 1:**
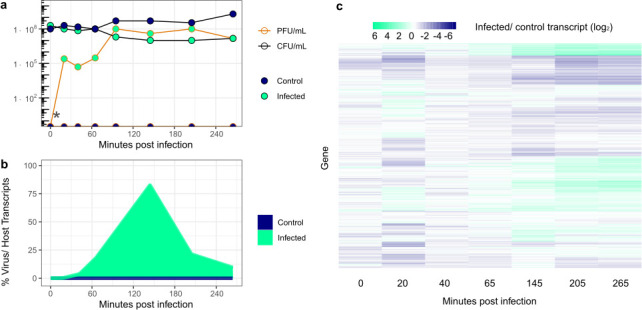
Global heatmap clustering during infection reveals bacterial response to infection. (**a**) Host growth measured by CFU and viral propagation measured via PFU. *At time 0, both cultures were measured before infection. Zero plaque-forming units were detected in the control (uninfected) condition, as expected (bottom of graph at 0). (**b**) Percentage of reads mapped to phage/host genome at each time point. Each value on the y axis is the median of technical triplicates. (**c**) Colors and intensity indicate ratio of median normalized counts comparing control and infection. Each row represents a gene in *B. cenocepacia* K56-2. Heatmap scale shown above image. This experiment was performed at an MOI of 3.

To establish a baseline of the infection kinetics and resistance profile of *B. cenocepacia* K56-2 and phage Bcep176*,* we set up a liquid infection. Samples for RNA, along with bacterial cell counts (CFU) and phage plaquing units (PFU), were taken over a 4-h time course of infection. *B. cenocepacia* K56-2 had a 100× reduction in bacterial cell concentration compared to control from 95 to 265 min post-infection (Fig. 1a). An increase in PFUs up to 1 × 10^8^ PFUs mL^−1^ was also observed starting at 95 min (Fig. 1a). The ratio of viral to host transcripts peaked above 80% at 145 min post-infection (Fig. 1b). The transcription of most *B. cenocepacia* K56-2 genes reached a relatively stable transcriptional profile beginning in late infection—starting at 145 min and lasting through 265 min post-infection (Fig. 1c). The steady PFU counts and an unchanging transcriptional response indicate *B. cenocepacia* K56-2 acquired resistance through Bcep176 lysogeny (Fig. S1) or additional mechanisms. Since it appeared the host acquired resistance within our experiment, we next assessed the predicted phage defense system landscape.

### Computational prediction and transcriptional validation of phage receptors and defense systems

We computationally predicted defense systems, common phage receptor genes, and genomic islands (see Materials and Methods) to understand the genomic landscape for phage–host interactions in *B. cenocepacia* K56-2. Altogether, we predicted 12 defense systems in the host *B. cenocepacia* K56-2, six defense system candidates, and six putative phage receptors throughout the genome. Additionally, we predicted eight genomic islands (two prophages, one integrative conjugative element, and five of unknown function) (Fig. S2 and Table S1).

**Fig 2:**
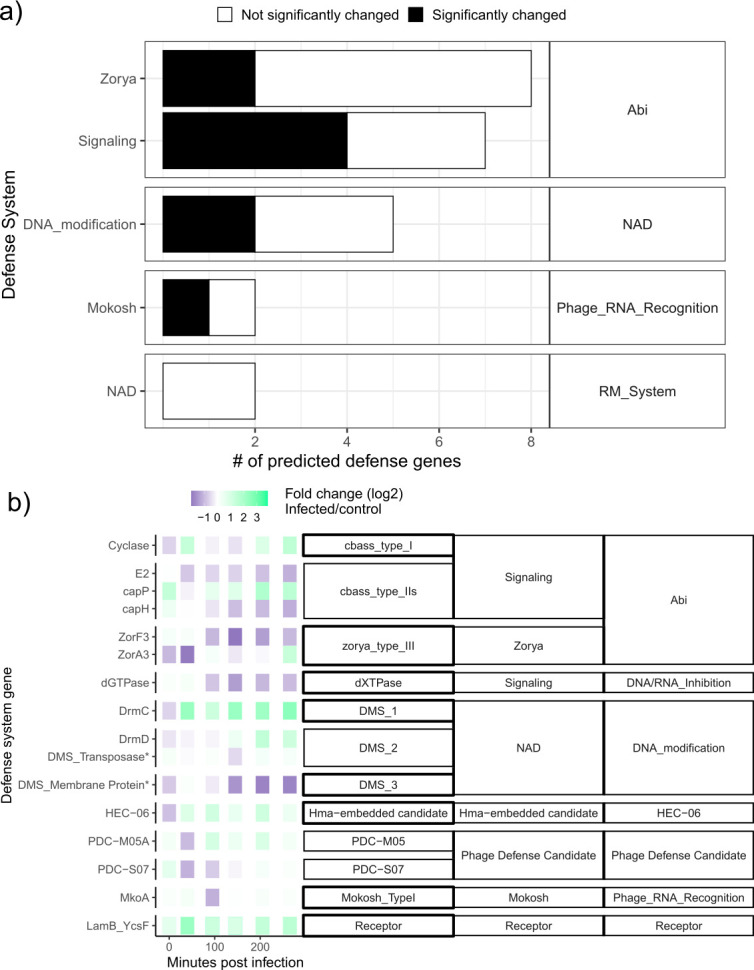
Viral infection significantly alters transcription of 39% of predicted defense systems. (**a**) Proportion of predicted defense genes significantly changed throughout the course of infection with Bcep176. Overall, the transcription of 39% of predicted defense gene was significantly altered in response to infection. (**b**) Genes within defense systems with significantly altered transcription in response to infection. Each row in represents one gene. Each box represents a time point. The transcriptional profile of every predicted gene, whether significantly changed or not, is shown in Fig. S3 formula).

We used phage-infected host transcriptomes to assess if computationally predicted defense systems were transcriptionally upregulated against Bcep176. Nine predicted defense systems were significantly changed over the course of infection (Fig. 2), with nine upregulated genes and eight downregulated genes during infection (Fig. 2b). For defense systems consisting of operons, either the entire operon was significantly changed in response to infection (Mokosh, PDC) or just the first one to two genes within the operon (Zorya I and Zorya III) (Fig. S3). For defense systems existing as single genes, systems that were changed were mainly upregulated by late infection (Fig. S3). Based on the transcriptional upregulation of the defense loci in *B. cenocepacia* K56-2 activity for PDC-M05A, HEC-06, DISARM protein DrmD, and CBASS Type I, we consider these to be transcriptionally active defense systems in this phage–host combination.

Six putative phage receptors were predicted using HMM searches against known phage receptors. Two LamB receptor gene homologs increased transcription compared to uninfected cells over the time course (Fig. S3), while the other predicted receptors remained statistically unchanged compared to control bacteria. Bacteria are known to express proteins at the membrane interfaces to prevent phage infection (34), so it is possible this change in expression may represent the host attempting to limit phage reinfection by changing membrane properties. Alternatively, LamB is a maltose transporter in *E. coli* (35); it is possible *B. cenocepacia* K56-2 cells increased their maltodextrin uptake in response to phage infection stress.

### CRISPRi screens reveal new phage resistance gene candidates and host factors required for productive phage infection

**Fig 3:**
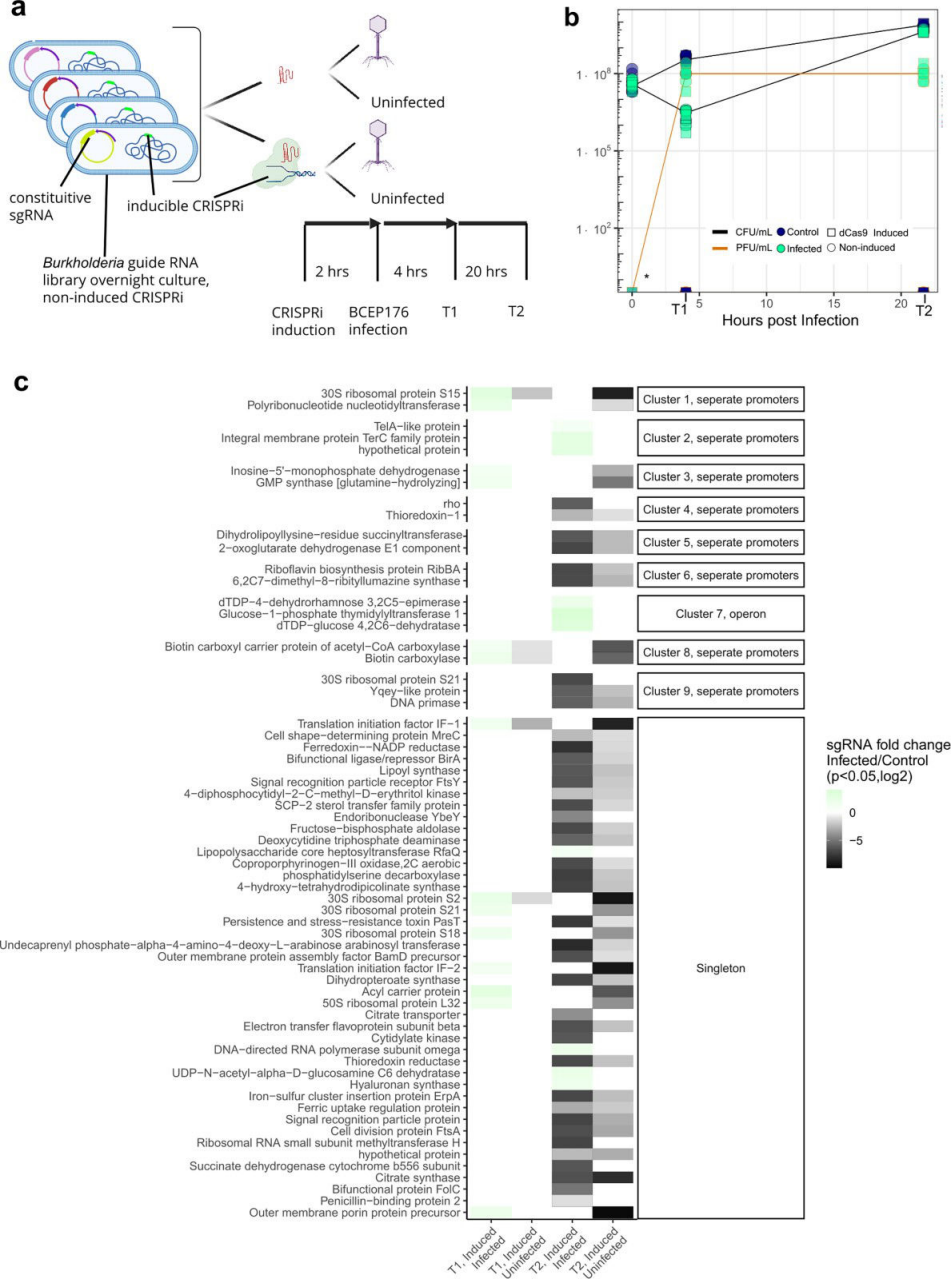
Genome-wide dCas9 screen reveals 65 novel candidate genes/gene clusters acting as phage host factors or phage resistance genes during phage infection. (**a**) Experimental setup of guide RNA library screen in *B. cenocepacia* 56-2. Experimental design of dCas9 screen. Guide RNA plasmid library with inducible dCas9 integrated into the genome of *B. cenocepacia* is grown overnight, aliquoted into four treatments, and introduced to Bcep176 phage infection (MOI = 3). (**b**) Quantification of phage and bacterial growth during CRISPRi experiment at each time point. At each time point, triplicate cultures were miniprepped, the guide RNA portion of their plasmid was sequenced, and relative abundance was compared between time points and experimental conditions. A placeholder value was used to graph the points where PFU/mL was zero in the uninfected control condition and at T = 0. (**c**) Heatmap of novel host factors and resistance genes identified in this screen. Each row represents one gene that was significantly altered during this experiment. The first and third columns indicate the change of relative abundance of guide RNAs in induced CRISPRi cultures, between infected (numerator) and control (denominator) cultures. A positive value indicates a host factor (green box in first or third column), while a negative value indicates a resistance gene candidate (dark gray box in first or third column). The second and fourth columns represent the change in guide abundance while cultures were uninfected between induced CRISPRi (numerator) and uninduced CRISPRi (denominator). A negative value (dark gray box) indicates an essential gene candidate.

The transcriptomic data provided from this time course provides a comprehensive view into changes in the host induced by viral infection but does not clearly determine the effect of each gene on host fitness. To this end, a CRISPRi-mediated knockdown screen was performed. Utilizing a previously developed rhamnose-inducible dCas9 system for *B. cenocepacia* K56-2, a genome-wide library was constructed (≤6 guide RNAs per gene, targeting 125 base pairs up and downstream of the start codon for each gene in *B. cenocepacia* K56-2) and infection experiments were performed taking time points to observe late infection (6 h post-infection) and natural resistance (24 h post-infection) (Fig. 3a and b). Guide RNA plasmids were extracted from each of these two time points and the relative abundance of guide RNAs was determined from each experimental condition (infected, CRISPRi induced, Fig. 3a). Genes targeted by guide RNAs that were significantly enriched in CRISPRi-induced, infected cultures compared to the CRISPRi-induced, uninfected control cultures were considered candidates for host factors, while genes targeted by guide RNAs that were depleted were considered candidates for phage resistance genes (see Materials and Methods for fitness).

**Fig 4:**
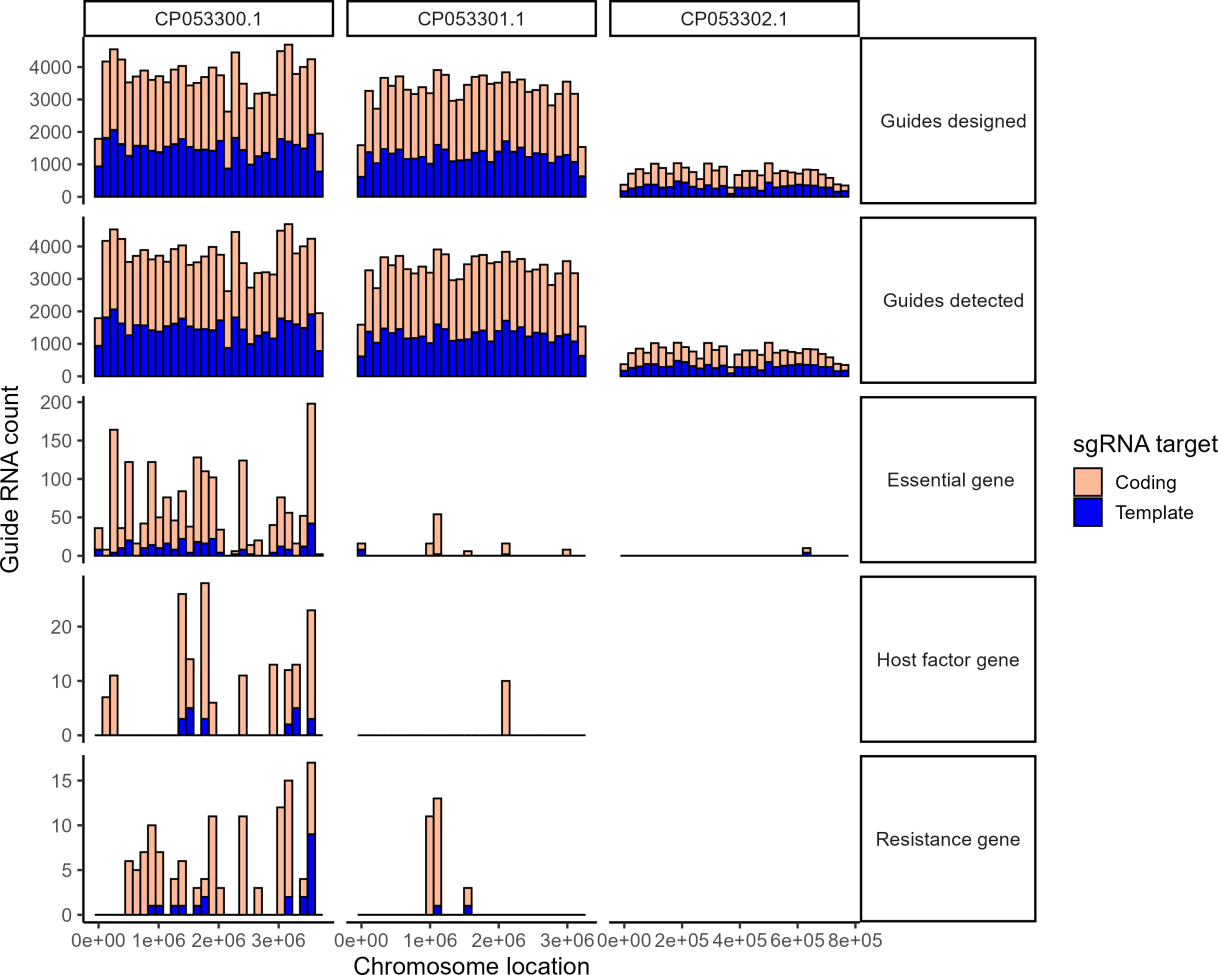
A majority of candidate essential genes, host factors, and resistance genes identified in this screen were located on a single chromosome. Chromosome, guide RNA strand targeted, and chromosome location split into panels—as designed, every guide detected by sequencing during this experiment, and those with fitness score of at least 3, categorized as essential genes, host factors, and resistance genes (see Materials and Methods for more detail on fitness classifications). Y-axis denotes count of individual guide RNAs found in each condition.

Between the two time points sampled during Bcep176 infection, collectively 65 genes had fitness scores of at least 3 (≥3 unique guide RNAs with same-direction fold change >|2|, adjusted *P* value <0.05) and were considered as host factor or resistance gene candidates (Fig. 3c; Table S2; see Materials and Methods for full explanation of candidate cutoffs). Among these candidates, eight gene clusters were identified (Fig. 3c), the rest were single genes (Fig. 3c). *B. cenocepacia* K56-2, like other species in the *Burkholderia* genus, has three chromosomes (33), and most essential genes and phage resistance and host factor candidates identified in this screen were located on the first chromosome, CP053300 (Fig. 4). Only one essential gene (annotated only as “hypothetical protein”) and no host factors or resistance genes were found within the third chromosome, CP053302. Guide RNAs were capable of knocking down host factors, resistance genes, and essential genes on both strands of DNA—coding and template—with 101 guides on coding strands and 605 on template strands (Fig. 4), which agrees with prior work in this strain (25). We found 148 genes that affected host growth in uninfected cultures but showed no difference during infection; these were considered essential genes (Table S2). None of the host defense systems predicted from annotation altered host fitness when knocked down during infection, even though their transcriptional changes were evident (Fig. 2). However, we found that some of our novel phage resistance candidates appeared to cluster with previously predicted defense genes (Fig. S1), suggesting that they may be related in function.

### Validation of candidate host factor and resistance genes

**Fig 5:**
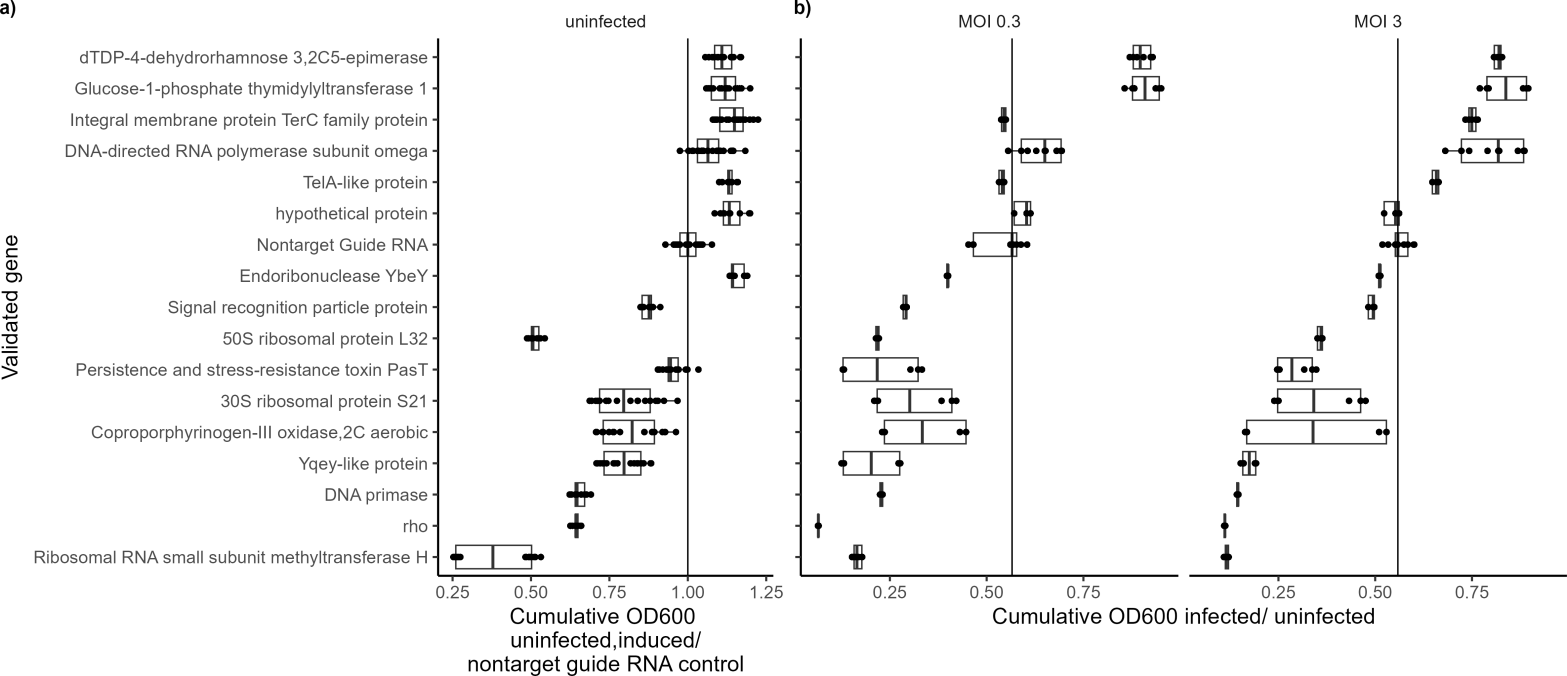
Validation of host factors and resistance genes*.* (**a**) Growth (OD600) at 24 h of induced single-guide knockouts compared to non-target guide control. Black vertical line (x = 1) is the median growth of non-target guide RNAs across all experimental days. Circles shown are replicates of guide RNAs grown in individual wells in a 96-well plate. (**b**) Growth (OD600) at 24 h of infected cells compared to respective non-infected controls of each guide RNA knockdown. Black line in each panel (~0.55) represents the growth of non-target guide RNA controls at 24 h. Single guides that had a higher proportion of growth than the non-target guide RNA knockdown are considered host factors, while guides with a lower proportion of growth are considered resistance genes.

To validate candidate host factors, single guide RNA knockdown strains were constructed, as well as a non-target guide RNA control, and a liquid assay using the same time points as the pooled genome-wide CRISPRi screen. Fifteen out of 16 knockdowns tested at MOI 3 (the same MOI used in the initial screen) resulted in the same enrichment or depletion of cell growth as first measured in the genome-wide screen (Fig. 3 and 5). It was observed that many of the host factors had higher growth rates after 24 h than the non-target guide RNA, and that many of the resistance genes had lower growth rates (Fig. 5a) based on OD600 readings. This could imply that knockdowns of host factors in this screen simply grew faster than wild-type cells, and thus were identified as being resistant to phage infection. Likewise, it could imply that resistance genes identified simply make cells grow slower and thus are identified as making the cell more susceptible to phage infection. The growth curves of each knockdown (Fig. S4) show that the differences in growth and death are more pronounced in these host factor and resistance gene candidates.

**Fig 6:**
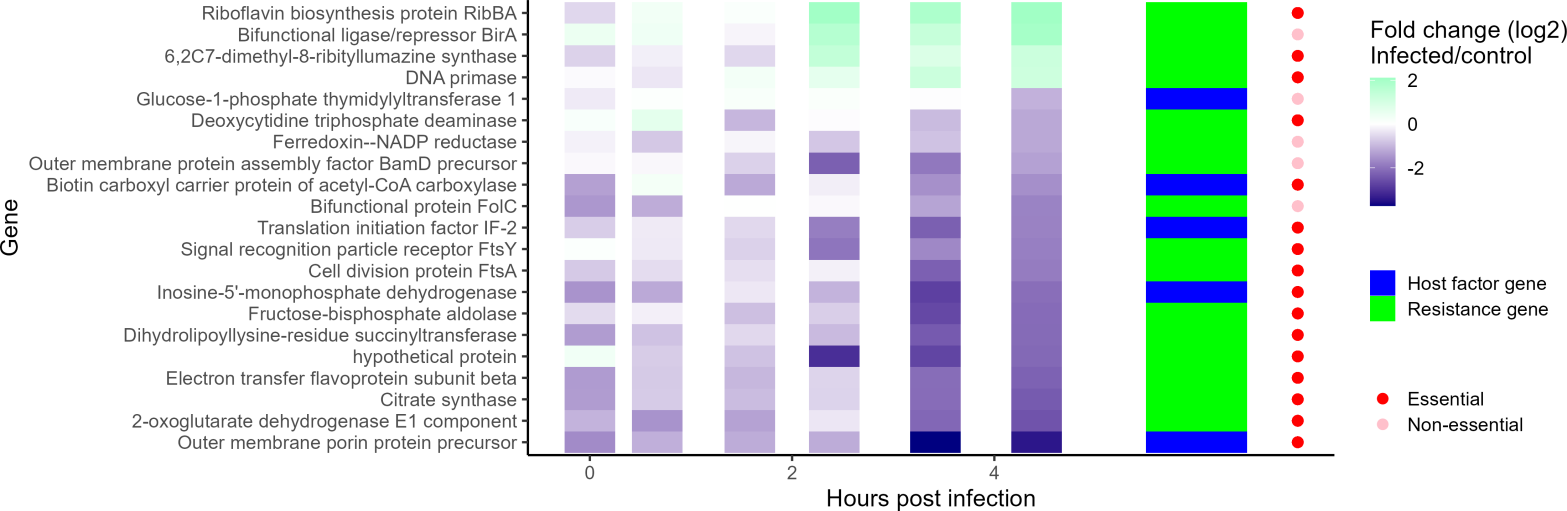
Infection significantly downregulates host factors and alters resistance gene expression. A subset of the host factors and resistance genes identified in this study is shown, which were significantly changed in transcription over the course of infection. Each row represents the transcription of a candidate host factor relative to uninfected cells. Each gene represented was significantly changed from the uninfected host in at least one time point throughout the course of infection and had a |fold change| >2 in the last time point, implying a role in late infection resistance. Columns on the right are colored by whether a candidate gene was a resistance or host factor candidate, whether the gene was deemed essential.

Since the CRISPRi screen and baseline RNA-sequencing data were both conducted in liquid, the transcription of the candidate host factor and resistance genes was analyzed within the context of native infection. All of the candidate host factor genes identified in this study were downregulated by late infection, while candidate phage resistance genes were split—some were up-regulated, and some were downregulated (Fig. 6). None of the candidate host factor genes, resistance genes, or essential genes (candidates from our screen and previously annotated) identified by the CRISPRi screen were found in the top 10 up or downregulated genes during infection (Fig. S5; Table S3). This may reflect that the phage is well-adapted to the bacterial host. Nonetheless, many candidate essential genes were downregulated by late infection, suggesting a strategy of slowing down metabolism or programmed cell death by the host. A group of four phage resistance gene candidates was significantly upregulated by the end of infection (Fig. 6, top four rows). These resistance genes include the gene annotated as DNA primase, which was validated separately. All the transcriptionally active host factor genes identified in this screen were downregulated by late infection, including the validated lipopolysaccharide (LPS) synthesis gene annotated as “Glucose-1-phosphate thymidyl transferase,” indicating another layer of defense against phage reinfection mediated on the transcriptional level.

## DISCUSSION

Here, we report an in-depth study of phage–host genetic interactions in *B. cenocepacia* K56-2. Overall, this screen identified 24 novel host factors and 41 phage resistance gene candidates (Table S2), many found in similar screens during model *E. coli* phage infection (36). We predicted numerous phage defense systems in this host which may prevent phage infection. We used a heterologous *E. coli* system to validate a CBASS Type IIs system that was predicted (Fig. S6). We chose not to further validate in heterologous hosts because we found four out of eight defense systems were not able to be cloned into *E. coli* host cells with a constitutively expressed promoter, assumed as cytotoxic, and because the focus was to discover novel phage–host interaction networks in *B. cenocepacia*. This work establishes a framework of experimentally validated phage–host interactions within the *Burkholderia* genus and informs future phage engineering/rebooting and phage therapy efforts against BCC bacteria.

While diverse candidate genes were validated as host factors or resistance genes, we noticed some emergent trends. Some of the most highly abundant host factors identified in our CRISPRi screen were involved in ribosome biogenesis, LPS biosynthesis, and quorum sensing, based on Kyoto Encyclopedia of Genes and Genomes (KEGG) enriched pathway analysis (Table S2). We observed that all annotated ribosomal genes were either unchanged or significantly downregulated in response to infection (Fig. S7). This late shift to reduce ribosomes may be a strategy by the host to inhibit phage protein production or globally repress translation to place the bacterial cell into a programmed cell death pathway. It may also be a strategy by the phage to stop host mRNA translation, freeing up resources for phage replication. It is known that some phages, but not Bcep176, encode their own ribosomal proteins as a strategy to promote their own replication late in the infection cycle (37), while other phages express genes that immediately co-opt host ribosomes (19), hijacking host translational machinery.

The next most common highly abundant gene found was “dTDP-L-Rhamnose biosynthesis,” which contained one member—“Glucose-1-phosphate thymidylyl transferase” which was significantly upregulated in response to infection (Fig. 6). It’s been found that these genes are critical for binding of phages to the LPS in *Listeria* (38, 39) and *Pseudomonas* (40). The role of LPS as a common phage receptor has been well documented (41–43). Finally, we identified quorum sensing genes as phage resistance candidates in our CRISPRi screen. These genes have been shown to help protect bacterial populations, especially those in microbial communities with high cell density, from subsequent phage infection (44) and help control lysis-lysogeny transitions (45).

At the onset of this study, we sought to validate computationally predicted phage defense systems in *Burkholderia* using our study system. However, none of the predicted defense systems (Table S1; Fig. S1) were identified in our CRISPRi screen as active defense systems, although 3 of the 34 computationally predicted individual defense system genes had less than three guide RNAs due to design constraints, disqualifying them to be significantly changed based on our criteria (see Materials and Methods). However, of these three computationally predicted defense system genes with limited guides, no guides were significantly changed during infection. Furthermore, none of the guides of the rest of the 31 predicted defense genes were significantly changed in abundance during our CRISPRi screen, strongly supporting the lack of defense activity detected in this screen. The explanation for this is fourfold: (i) the predicted defense systems are not active against Bcep176 since a productive infection occurs. (ii) The defense systems are active at low copy number, and knocking their mRNA production down for 2 h prior to infection was not sufficient to alter their host’s fitness during infection. (iii) *Burkholderia* is phylogenetically distant from most well-characterized and studied systems *E. coli*, *B. subtilis,* and *Salmonella* spp*.* (16, 46); therefore, the profiles used are too dissimilar to allow for detection of defense systems. (iv) *Burkholderia* is well known to have genome plasticity (47–49), which causes large portions of the *Burkholderia* genome to be unique in comparative analysis (49), so there may be undetected novel defense systems which did not happen to activate with this phage.

We compared the novel host factors and resistance gene candidates from our screen to recent studies which used CRISPRi screens to identify host factor and resistance genes in *E. coli* (36, 50). We found 28 phage resistance genes identified in our studies active against the Coliphages tested (Lambda and Coliphage 186, two model temperate phages infecting *E. coli*, and T4 and T7, model lytic phages). Overall, more resistance genes were shared with Lambda and 186, likely suggesting temperate and lytic phages trigger different resistance gene activation across broad hosts. However, relatively few host factors (~17) were found in the *E. coli* screen of these phages. This likely highlights the difference between phage infection in diverse host strains and may partly reflect the differences in lipopolysaccharides between *E. coli* (51) and *Burkholderia* species (52). The unique host factors found in the present study have potential to be utilized for phage engineering efforts. Previous work has shown phage host factors, such as rare tRNAs (53) and sigma factors (20), increased the ability to reboot naked phage DNA in heterologous systems.

A key limitation of our CRISPRi screen is likely due to *Burkholderia’s* high rate of mutation and recombination (54–56). The largest single comparison of significantly changed guide abundances was the uninduced infected vs control at 24 h post-infection (Fig. S8). While these large differences of uninduced guide sequences may be a sign of leaky expression, the sharp drop-off from three guide hits/gene to four in the uninduced infected vs control suggests that these are likely background mutations within the genome of cells harboring these guide RNAs. To avoid the potential impact these mutations could have, we filtered out any guide sequences that were significantly changed in uninduced cultures before calculating fitness score to determine novel host factor and phage resistance gene candidates.

The impact of lysogeny on high-throughput screens is not typically analyzed but may be a factor contributing to the observed resistance to phage infection during the screen. The final time point was 24 h post-infection during the CRISPRi screen, during which both induced and non-induced CRISPRi populations recovered from the initial crash, but phage concentration remained constant (Fig. 3b). This suggests phage resistance was acquired; we sought to further understand if this was due to the CRISPRi or Bcep176 integration. We identified the *attB* site in *B. cenocepacia* K56-2 and tested if Bcep176 was integrated at either time point of the CRISPRi screen using PCR. Lysogeny does occur at the 24 h time point in 5/6 of the replicates tested but only occurred in a single replicate tested at 4 h (Fig. S1). Additionally, all replicates tested positive for a non-lysogenic state (Fig. S1). This suggests that the resistance observed may be partly due to Bcep176 repressor-mediated immunity of a portion of the population. This could potentially be verified by transcriptomes at a later time point (our latest transcriptome time point was ~4.5 h post-infection), but due to multiple rounds of infection, this signal would likely be difficult to discern without single-cell resolution, which is outside the scope of this study.

Overall, we unveiled many novel genes associated with phage–host interactions in *Burkholderia*. These genes could serve as new targets for engineered phage therapeutics to combat difficult-to-treat *Burkholderia* infections and inform *Burkholderia* phage rebooting efforts. Additionally, these genes could be useful for understanding barriers to *Burkholderia* genome engineering. However, as typical with high-throughput screens, more questions are evident and require future investigations. Some of these include (i) what are the roles of ribosomal proteins in regulating phage infection? (ii) How to predict and validate defense systems in hosts phylogenetically remote from model systems? This work provides approaches that phage researchers may find of value in addressing these questions.

## MATERIALS AND METHODS

### Bacterial and phage strains used in this study

*B. cenocepacia* K56-2 (GenBank assembly: GCA_014357995.1), originally isolated from sputum from a cystic fibrosis patient (33), was obtained from BEI (NR-20535) and used in this study. For all *B. cenocepacia* K56-2 infection experiments, Luria Broth (LB) media was used, and cells were grown at 37°C and shaken at 200 RPM.

### Bcep176 phage induction and isolation

*B. multivorans* ATCC17616 prophages were induced using mitomycin C (MMC). An overnight culture of *B*. *multivorans* ATCC17616 was grown in LB broth at 37°C shaking, diluted to an OD600 = 0.02 in LB broth, and incubated at 37°C shaking until the OD600 reached 0.5. MMC (100 ug/uL) was added, and samples were taken 2 h post-MMC. Samples were centrifuged at 16,000 × *g* for 2 min and filtered using a 0.2 µm syringe filter. Filtrates were spot tested on *B. cenocepacia* K56-2 lawns, plaque purified three times, and scaled to a high titer lysate for sequence verification and further experiments. The prophage was sequence confirmed to be Bcep176 (AP009386.1: 1620491-1665337) infecting *B. cenocepacia* K56-2 (previously discovered and sequenced NCBI accession: NC_007497.1).

### Bcep176 phage infection time course

On the day of infection, overnight *B. cenocepacia* K56-2 cultures were diluted into two glass flasks to an OD600 of 0.005 and grown to an OD600 of 0.03. At OD 0.03, cells were infected at an MOI of 3, while an equivalent volume of phage buffer was added to the uninfected control. Aliquots for RNA sequencing, CFUs and PFUs were taken pre-infection (0 min), and at 20, 40, 65, 95, 145, 205, and 265 min. Aliquots for RNA sequencing were placed in RNAProtect Bacteria Reagent (Qiagen) and stored at −80°C. Cultures at each time point were plated on LB plates for CFUs and filtered (0.2 µm) and spotted on bacterial lawns to determine PFUs.

### RNA sequencing

RNA was extracted from preserved aliquots using RNEasy kit (Qiagen) and DNA was removed with Turbo DNA-free kit (Qiagen). cDNA was synthesized and libraries were prepared with TruSeq library Prep with RiboZero (Illumina). Technical triplicates were prepared from each time point. cDNA libraries were run on the Illumina Nextera 550 using single-end 75 bp sequencing kit. Raw sequencing data were mapped to phage and bacteria genomes using Bowtie2 (version 2.4.1) (57). Samtools (version 1.13) (58) was used to convert between file formats for downstream analysis.

### Differential expression analysis

HTSeq (version 0.11.3) (59) was used to count the number of reads per annotated gene product in both the bacterial genome and the phage genome, and these read counts were used as read counts for downstream differential expression analysis. The 95-min time point was left out of further transcriptomic analysis due to two of the triplicate samples with zero reads per gene (88%, 73%, of genes had zero reads compared to the median of 6% of genes with zero reads for all other samples). Differentially expressed genes were determined with DeSeq2 (version 1.40.2) (60) using the Likelihood Ratio Test (LRT, adjusted *P* value < 0.05) with a design of time and infection status, with a reduced model of infection without time. This approach considers differences between bacterial cultures pre-infection (0 min) and identifies genes expressed at different slopes over the course of the experiment. Heatmaps and other analyses were performed in RStudio (4.3.0). Medians of normalized read counts of differentially expressed genes were used for visualization in the R package ggplot2.

### Computational prediction of defense loci, receptors, and genomic islands

Defense systems were predicted by padloc (46) for *B. cenocepacia* K56-2. CRISPR-Cas systems and spacers were predicted by CRISPR-Cas Finder (61).

We developed custom HMMs for known phage receptor proteins: GamR (62), TonB (63), FepA (63), FhuA (64), BtuB (65), TolC (66), OmpA (67), OmpW (68), OmpC (69), OmpK (70), FadL (71), BamA (72). We obtained reference protein sequences from the strain identified in each publication and used BLASTp (73) to obtain homologous sequences (>40% similarity). These proteins were aligned and HMMs were built using HMMER v3.1b2 (74) with default parameters. HMMs were tested against the originating genome and any with outputs scoring lower than 50, an e-value lower than 0.01, and less than 90% accuracy were removed.

Genomic islands were predicted using TIGER (75, 76) and Islander (77) software with default parameters.

### CRISPRi knockdown library construction

A codon-optimized dCas9 (CRISPRi) was cloned into *B. cenocepacia* K56-2 as previously described (25). pAH-CTX1-rhadCas9, pAH18, pAH25-SceI, and pgRNA-non-target were gifts from Silvia Cardona (Addgene plasmid # 129391, 154397, 129389, 129465, respectively). Guide RNAs were designed using the Guidemaker program (v.0.3.4) (78) using NGG at the 5′ position of the coding strand (dCas9). Twenty base pairs were chosen as the guide length, and features were selected for the lowest off-target cutting efficiency and proximity to the start codon based on NCBI’s annotation for the three chromosomes of this strain (NZ_CP053300.1, NZ_CP053301.1, NZ_CP053302.1). Both positive and negative strands were used as targets for each gene, as they were shown to be equally effective in silencing genes in *B. cenocepacia K56-2* (25). Guide library oligos were synthesized by Twist Bioscience and cloned into pgRNA-non-target vector backbones via restriction digestion/ligation. The resulting plasmid library was extracted from plated transformations at theoretical 20× coverage of 38,826 unique guide sequences, then cloned into *E. coli* MFDpir at a theoretical coverage depth of 20× and lastly conjugated into *B. cenocepacia* K56-2 cells expressing rhamnose-inducible dCas9 (25) at a theoretical coverage of 20×.

### CRISPRi knockdown library sequencing and differential abundance analysis

Guide RNA library plasmids were miniprepped (Qiagen) at 6 and 24 h post-infection with Bcep176. They were amplified with primers 466 and 467 (Table S4), PCR purified (NEB), and the resulting amplicon library was sequenced (Genewiz). The resulting reads were filtered for length and quality over 30, then the abundance of each unique contig was determined. Unique contigs from each sample were further filtered to include a 100% match to the J3119 (SpeI) promoter and the first 20 base pairs of the gRNA scaffold within pgRNA non-target. Plasmid-derived sequences were removed from the amplicon, resulting in 20 base pairs corresponding to guide RNAs. These guide RNA sequences were filtered to not include any ambiguous bases. When no reads were found corresponding to a guide RNA from a sample, a raw read count of zero was used. Guide RNAs that were within 125 base pairs of the start codon for genes were counted twice if applicable—i.e., if a guide RNA was in between the 5′ of two genes with opposite strand orientations, it was counted as active with both genes for differential expression analysis. The differential abundance of guide RNAs between three replicates of CRISPRi induced, uninfected and CRISPRi induced, infected was analyzed via DeSeq2 to determine the fold change and adjusted *P* value of significantly changed guide RNAs. If a guide RNA’s differential abundance was significantly enriched between infected and uninfected cultures (fold change >2, adjusted *P* value < 0.05) for the comparison made, the gene it was targeting received a fitness score of +1, while significantly depleted genes (fold change <0.5, adjusted *P* value < 0.05) received a fitness score of −1. Genes with fitness scores above 2 or below −2 were considered host factors and phage resistance gene candidates, respectively. Similarly, guide RNA abundance between induced and not induced with a fitness score of below −2 was considered essential genes. To remove the potential effect of background mutations in the host genome conferring resistance acquired during library construction passages, guide RNAs that were significantly changed (|fold change| > 1, adjusted *P* value < 0.05) between infected and non-infected of uninduced cultures were not included when determining fitness scores of each gene.

### Gene annotations and KEGG predictions

Gene annotations used in this text for genes that were not annotated as defense systems as described above were taken from GFFs for the three chromosomes of this strain found on NCBI (NZ_CP053300.1, NZ_CP053301.1, NZ_CP053302.1). Translated nucleotide queries of predicted coding sequences standard genetic code (code 11) were used to query EGGNOG mapper v2 (79, 80) KEGG Terms and Modules.

### Statistics

#### Differential expression in native infection

To determine if a gene was significantly changed throughout the course of infection, DeSeq2 differential abundance was implemented using the LRT with a design of infected + time + infected:time. To determine the fold change at each time point of infected compared to control for visualization purposes, DeSeq2 was utilized using the Wald test with a design of infected and repeated for each time point. To determine if a gene was significantly changed over the time course (LRT), or in each time point (Wald), a cutoff Benjamini-Hochberg adjusted *P* value of 0.05 was used.

#### Clustering of transcriptional fold change

Fold change data were used to create a matrix and then clustering using pheatmap’s (https://github.com/raivokolde/pheatmap) default values was used. Fold change values were taken from DESeq2 Wald comparing transcription between each time point (infected/controlled).

#### Differential expression of guide RNAs

DeSeq2 Wald test was used to determine the relative abundance fold change and *P* value of each guide sequence within time points 1 and 2. Comparisons were made between triplicates of each experimental condition (infection, CRISPRi induction). A cutoff Benjamini-Hochberg adjusted *P* value of 0.05 was used.

#### Significantly changed efficiency of plating

To validate if a strain with a knockdown of a candidate host factor was significantly changed, the PFU/mL of triplicates of a knockdown was compared against triplicates of the non-target guide RNA control. A one-sided *t*-test (hypothesized that knockdowns with and without inducer would produce less PFU/mL than non-target control) was performed and a cutoff Benjamini-Hochberg adjusted *P* value of 0.05 was used. Median values of the fold change of PFU/mL were used for visualization. The equation used to calculate efficiency of plating (EOP) is the following equation. EOP = (median PFU/mL targeted knockdown)/(median PFU/mL non-targeted control).

### Heterologous expression of predicted *Burkholderia* defense systems in *E. coli*

The DNA sequences of predicted systems were amplified using *B. cenocepacia* K56-2 genomic DNA as a template, cloned into pAYC184 (replacing the Tetracycline resistance gene), and expressed constitutively in *E. coli* MG1655. Inserts were confirmed via Sanger sequencing. Lawns of *E. coli* strains expressing defense systems were challenged with T4, T5, T7, Lambda, and P1 phages via a spot test and compared to a control *E. coli* with an empty vector to determine whether the heterologously-expressed genes conferred resistance.

## Data Availability

RNA-sequencing and CRISPRi sgRNA amplicon raw reads are deposited at NCBI Sequence Read Archive (SRA) database. The raw sequencing can be accessed from BioSample SAMN45153462–SAMN45153509. All raw sequence data can be found under BioProject accession PRJNA1178717.

## SUPPLEMENTAL MATERIAL

Supplemental figures spectrum.01936-25-s0001.pdf Fig. S1 to S8.

Table S1 spectrum.01936-25-s0002.xlsx Genomic location of genomic islands and predicted defense systems.

Table S2 spectrum.01936-25-s0003.xlsx Genomic location of host factors, resistance genes, and essential genes.

Table S3 spectrum.01936-25-s0004.xlsx Top 10 upregulated and downregulated genes during wild-type infection of B. cenocepacia 56-2 with BCEP176.

Table S4 spectrum.01936-25-s0005.xlsx Primers used in this study.

ASM does not own the copyrights to Supplemental Material that may be linked to, or accessed through, an article. The authors have granted ASM a non-exclusive, world-wide license to publish the Supplemental Material files. Please contact the corresponding author directly for reuse.
