# Supplementary material for: Exploring phage–host interactions in Burkholderia cepacia complex bacterium to reveal host factors and phage resistance genes using CRISPRi functional genomics and transcriptomics
Source: Microbiol Spectr. 2025 Oct 2;13(11):e01936-25. doi: 10.1128/spectrum.01936-25 (PMC12584637; doi:10.1128/spectrum.01936-25)
Supplement: Supplemental figures — Fig. S1 to S8. [file spectrum.01936-25-s0001.pdf]

Supplementary Figures

Supplementary Table 1. Genomic locations of defense systems, receptors, and genomic islands for Burkholderia cenocepacia K56-2.

Supplementary Table 2. Genomic locations of essential genes, host factor candidates, and resistance candidates as identified in CRISPRi screens and KEGG annotations of host factor and resistance candidates

Supplementary Table 3 - Top 10 upregulated and downregulated genes during wild-type infection of B. cenocepacia 56-2 with BCEP176

Supplementary Table 4 - Primers used in this study

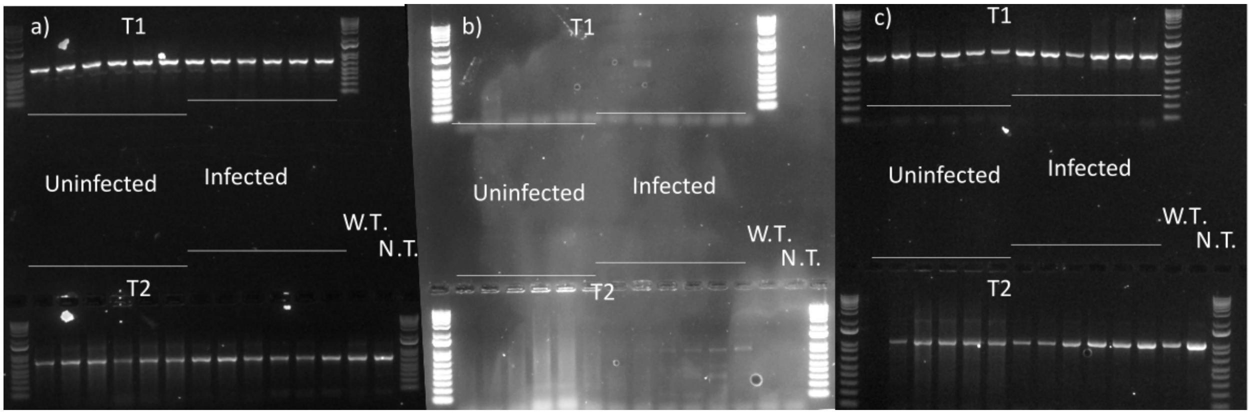

Supplementary Figure 1. A portion of the population during late infection underwent lysogeny with BCEP176 during CRISPRi guide library experiment. First and last lanes represent 1Kb+ DNA ladders (Invitrogen). All lanes represent samples diluted and subjected to colony PCR in the same order, each panel has different primer sets. The top row are from T1 (4 hours post infection) and the bottom row are from T2 (20 hours post infection). From left to right, lanes 1-3 are replicates of uninfected, uninduced cultures. Lanes 4-6 are uninfected, CRISPRi-induced cultures. Lanes 7-9 are infected, uninduced cultures. Lanes 10-12 are infected, CRISPRi-induced cultures. The last two lanes on the bottom row are uninfected controls – overnight cultures of wild type (W.T.) *B. cenocepacia* K56-2 and a *B. cenocepacia* K56-2 strain with the CRISPRi system installed and a plasmid expressing a nontarget guide RNA (N.T.) **A)** Housekeeping control – elongation factor IL-2. **B)** AttL – primers spanning the left integration sight (within “amino acid transporter” gene) and the left side of the phage *Bcep176*. **C)** Primers spanning the entire “amino acid transporter” gene with the predicted phage integration site. If *Bcep176* integrated, it would be too large to amplify, and no band would appear.

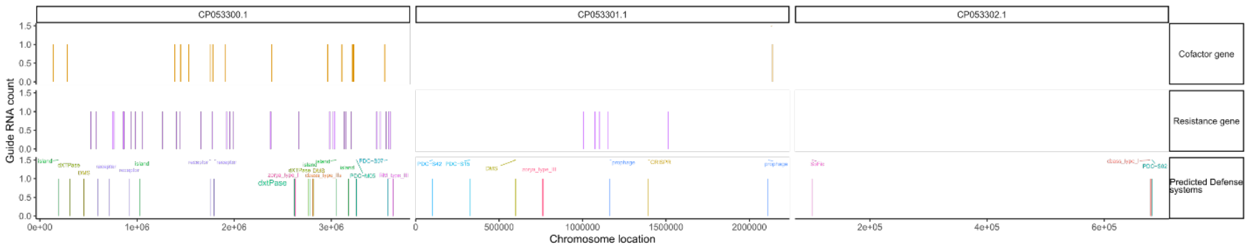

Supplementary Figure 2. Some phage resistance gene candidates identified in this study overlap predicated defense systems.

Different types of predicted defense systems are colored and labeled with their position on each respective chromosome in *B. cenocepacia* K56-2.

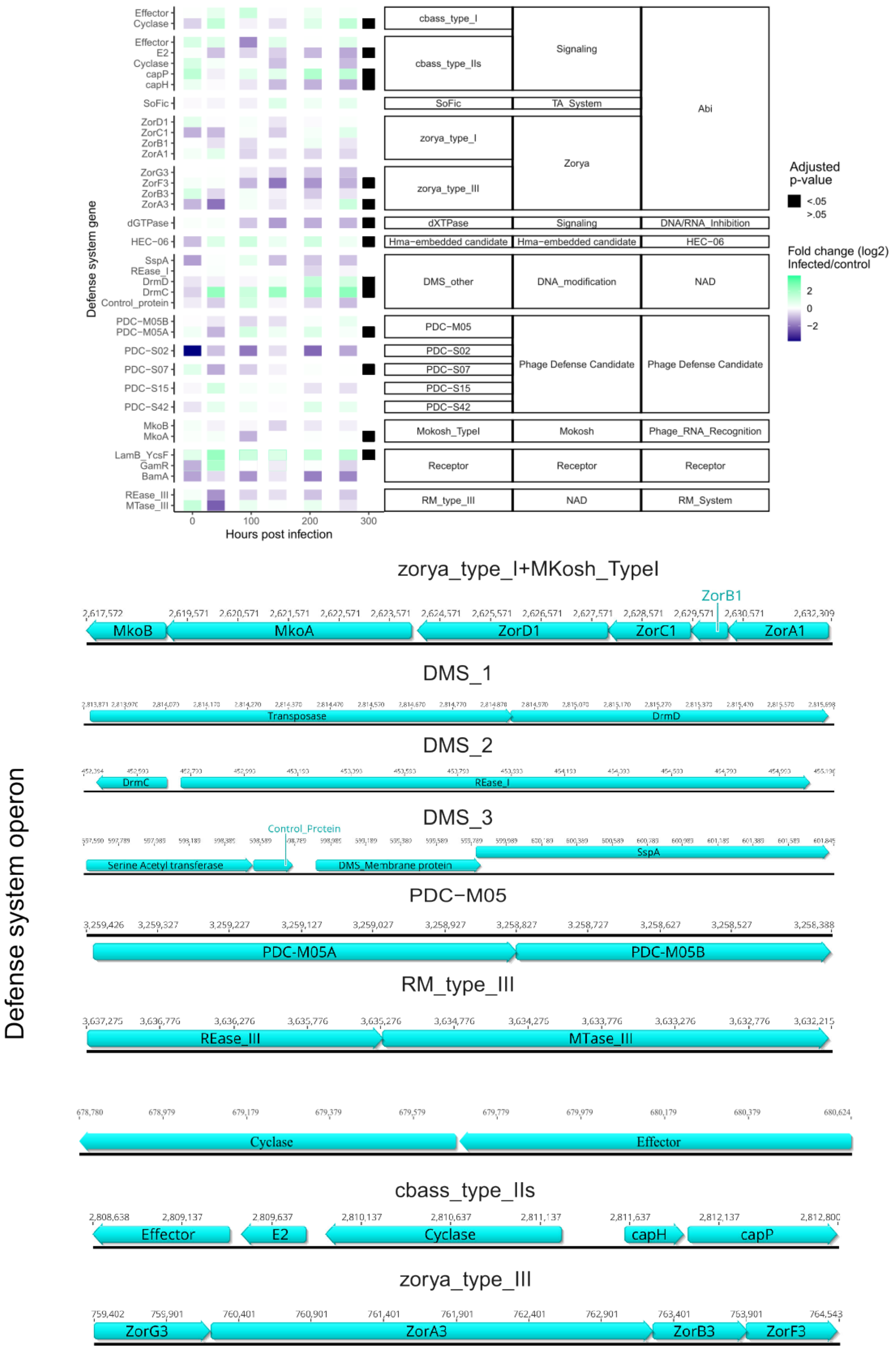

Supplementary Figure 3. Segments of defense system operon transcription changed in response to phage infection. Heatmap values are the fold change of the median of triplicate samples. The midpoint of each rectangle is the time of sampling. Each row represents a single gene found within a defense system operon. The black box or absence of black box represents genes that were significantly changed over the time-course of infection compared to uninfected

cells (LTR, adjusted *p* value<0.05). *Genes with an asterisk were not predicted as defense systems but share an operon with a defense gene.

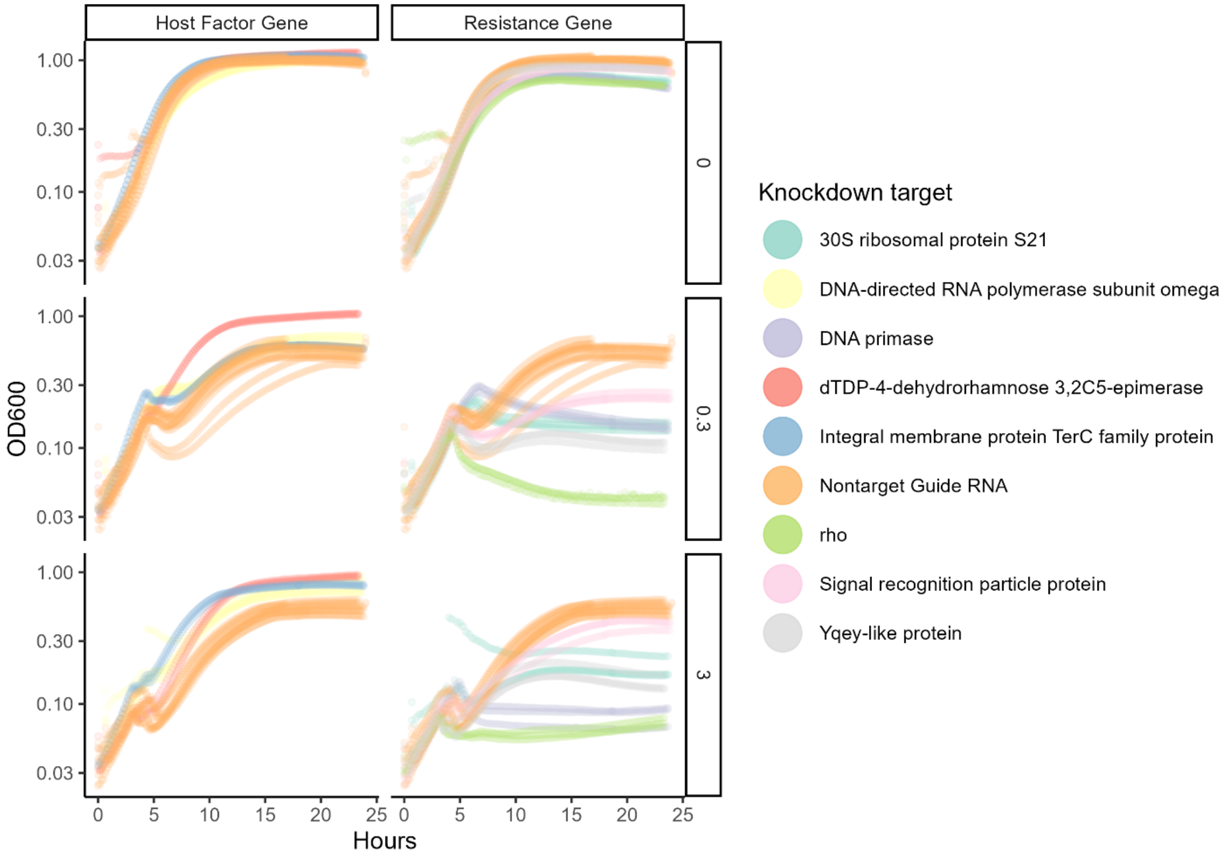

Supplementary Figure 4. Guide RNA's validation: knocking down host factor genes increases host fitness during infection and knocking down phage resistance genes decreases phage fitness during infection, while uninfected knockdowns of both are relatively unaffected. Points shown are colored by the target of the single guide RNA in each strain tested to validate the genome wide screen (**Figure 3)**. Genes were predicted as a host factor gene from the genome wide CRISPRi screen if they conferred resistance when knocked down, and were predicted as resistance gene from the genome wide CRISPRi screen if they made the cell more susceptible to phage infection when knocked down.

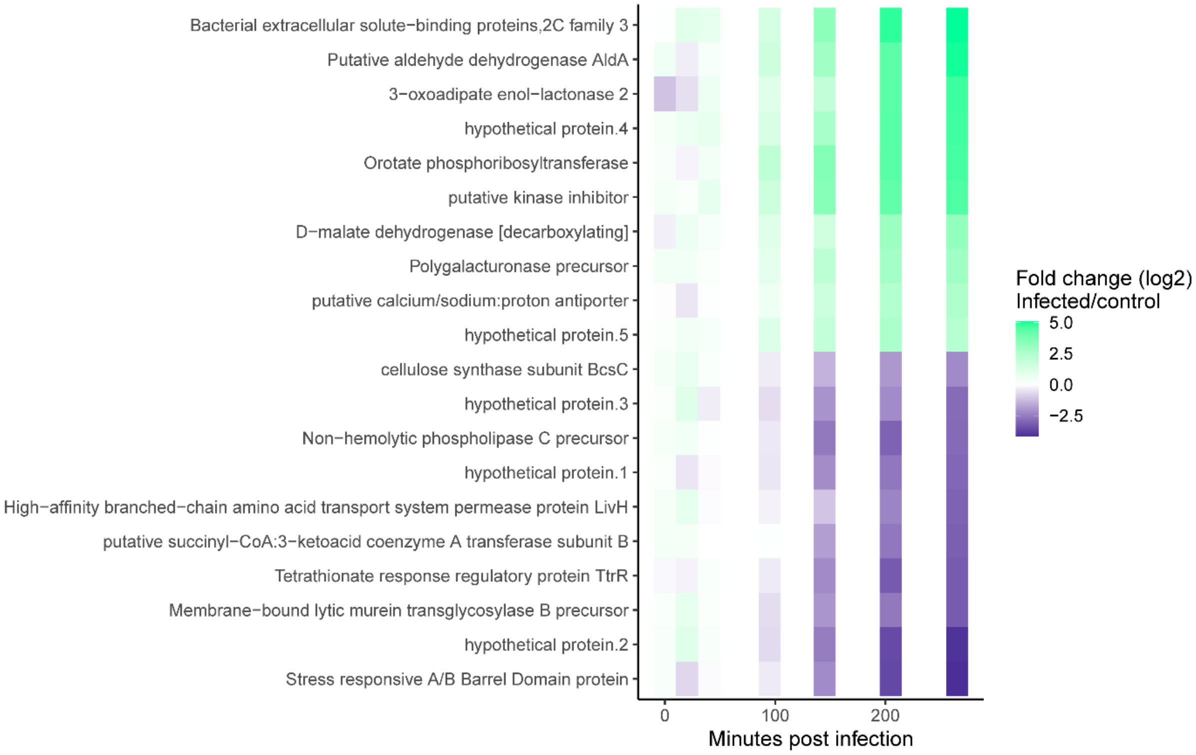

Supplementary Figure 5. Diverse proteins are highly upregulated and downregulated upon infection. Each row in the heatmap represents a gene, and the filling of each box represents the fold change of infected cells versus control cells. The top 10 up and down regulated genes were selected based on the final fold change value being <-2 or >2 and having lowest adjusted p-value of differential expression over time and infection (DeSeq2, LRT). Given that five proteins were identified as “hypothetical protein”, a label of “.1”, etc. was used to differentiate between these proteins.

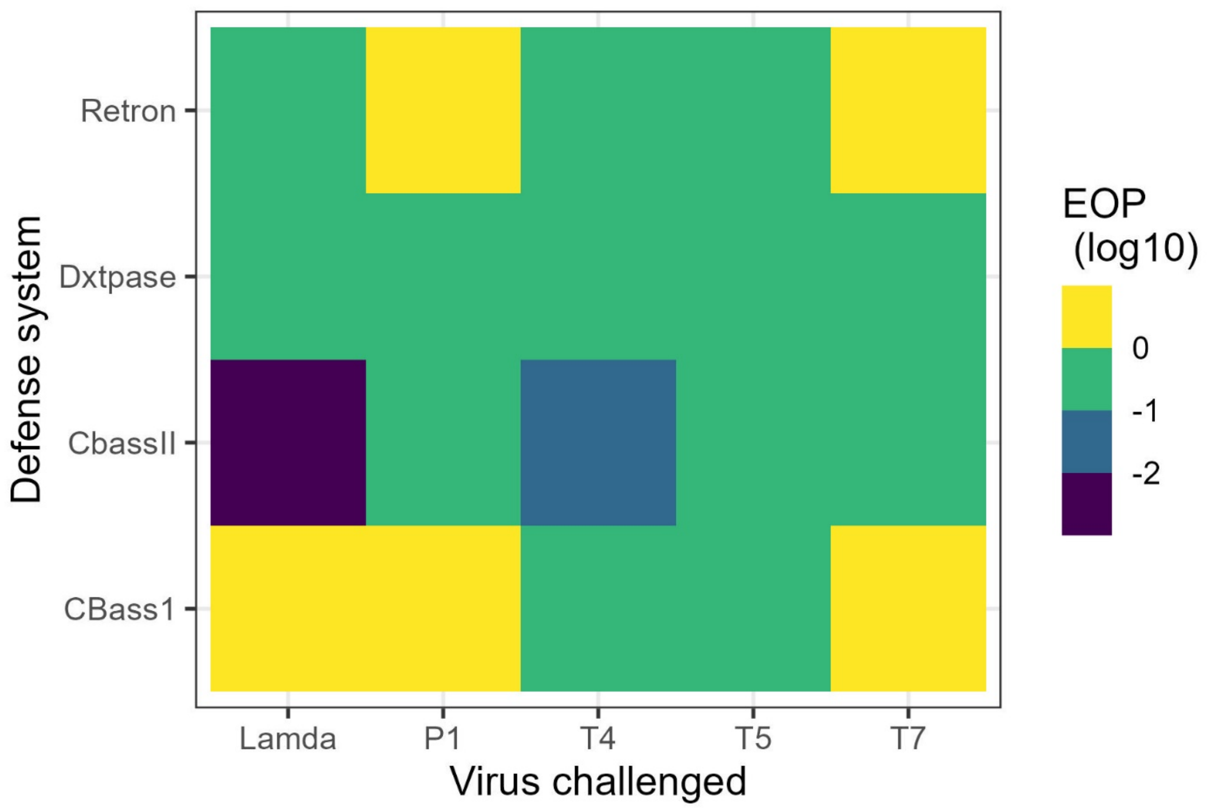

Supplementary Figure 6. CBASS II system from *B. cenocepacia* 56-2 heterologously expressed in *E. coli* MG1655 protects against infection from two coliphages. Entire defense operons were cloned into constitutive expression vector pACYC184. Heatmap is colored by the median value of the efficiency of plating (EOP), determined against empty vector control.

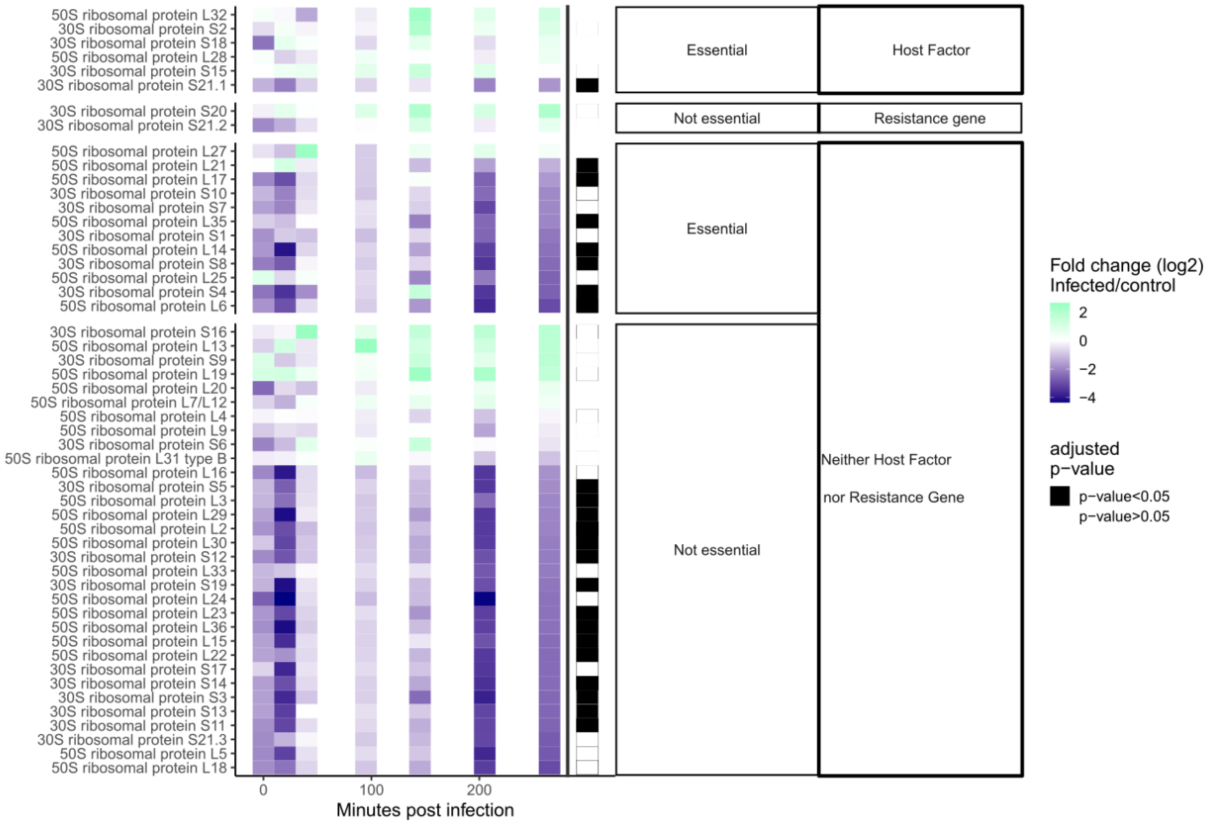

Supplementary Figure 7. Multiple ribosomal transcripts are significantly downregulated during phage infection.

Each row represents a unique gene annotated as a ribosomal protein. Since 3 proteins were annotated at “30S ribosomal protein S21”, unique variants were denoted as “.1”,”.2” and “.3”. The last column is colored by whether the gene was significantly changed over the course of infection.

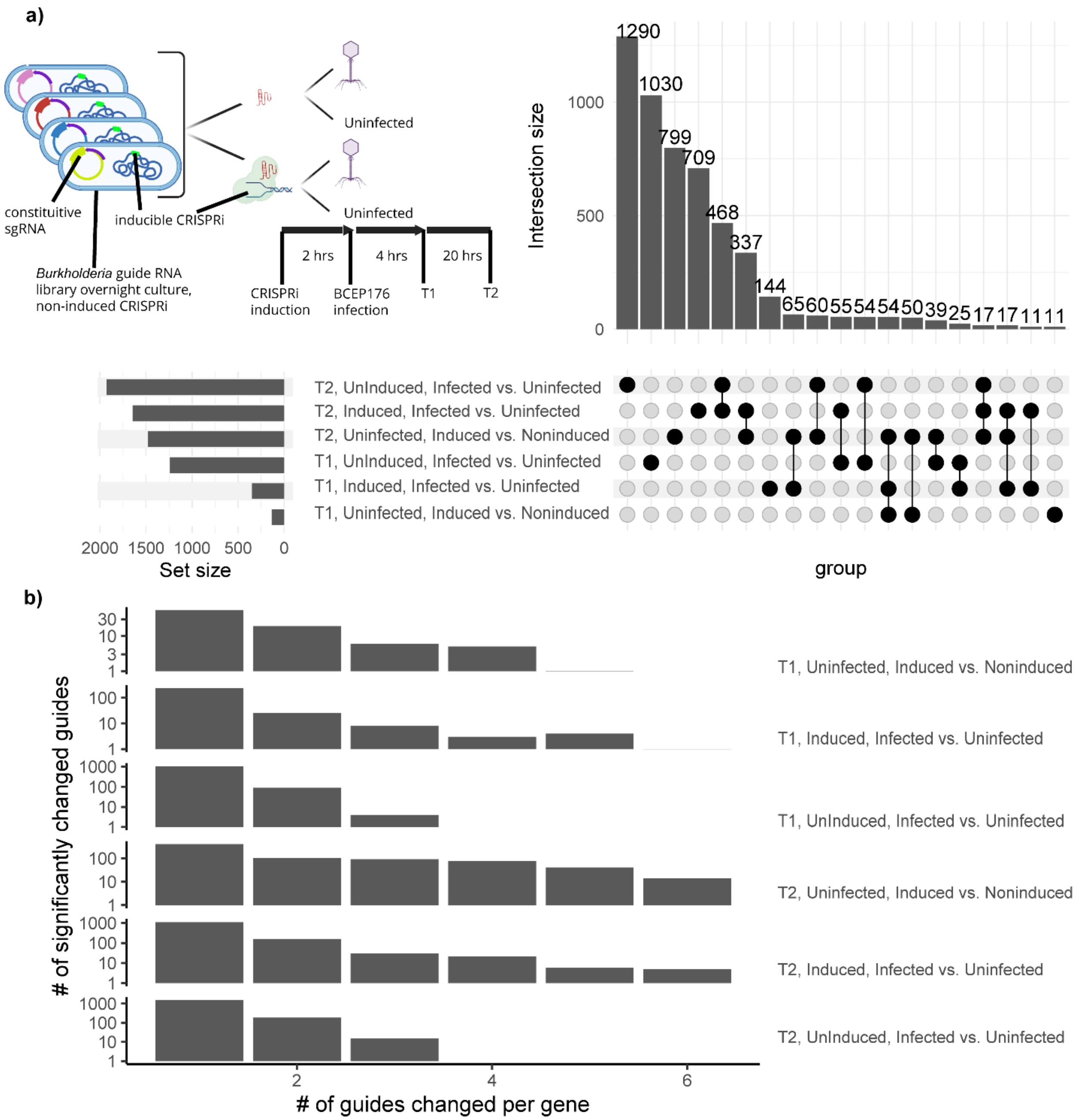

Supplementary Figure 8. Most significantly changed guide RNA's in CRISPRi screen from uninduced, infected vs. noninfected condition. **a)** Comparisons made during guide library screen along with key of experimental setup. Guides were considered significantly changed if they had an adjusted *p-value_ <0.05 and* a |fold change|>2. **b)** Data from **a)** parsed by matches to gene. Each gene had up to six guide RNAs designed. Note that the distribution of guide hits is closer to one hit per gene for "Uninduced, Infected vs. Uninfected" comparisons.
